# The contribution of *Pseudomonas aeruginosa* infection to clinical outcomes in bronchiectasis: a prospective cohort study

**DOI:** 10.1080/07853890.2021.1900594

**Published:** 2021-03-23

**Authors:** Rongchun Wang, Shuizi Ding, Cheng Lei, Danhui Yang, Hong Luo

**Affiliations:** a Department of Respiratory and Critical Care Medicine, The Second Xiangya Hospital, Central South University, Changsha, China; b Research Unit of Respiratory Disease, Central South University, Changsha, China; c Hunan Diagnosis and Treatment Center of Respiratory Disease, Changsha, China

**Keywords:** Bronchiectasis, *Pseudomonas aeruginosa*, mortality, hospitalizationsexacerbations

## Abstract

**Objectives:**

The impact of *Pseudomonas aeruginosa* on the prognosis of bronchiectasis remains controversial. This study aimed to explore the prognostic value of *P. aeruginosa* in adult patients with bronchiectasis in central-southern China.

**Patients and methods:**

This prospective cohort study enrolled 1,234 patients with bronchiectasis between 2013 and 2019. The independent impact of *P. aeruginosa* on all-cause mortality, annual exacerbations, and hospitalizations was assessed.

**Results:**

*P. aeruginosa* was isolated from 244 patients (19.8%). A total of 188 patients died over a follow-up period of 16 (1–36) months. Patients with *P. aeruginosa* had a longer disease course, poorer lung function, more lung lobe involvement, and more severe Bronchiectasis Severity Index (BSI) stage than those without *P. aeruginosa*. The independent impact of *P. aeruginosa* was observed on frequent hospitalizations but not on mortality and frequent exacerbations. Moderate- or high-risk comorbidities increased the risk of mortality (hazard ratio [HR]: 1.93, 95% confidence interval [CI]: 1.26–2.95), and this effect was magnified by the presence of *P. aeruginosa* (HR: 2.11, 95% CI: 1.28–3.48).

**Conclusions:**

*P. aeruginosa* infection acts as a marker of disease severity as well as predictor of frequent hospitalizations. *P. aeruginosa* had no independent effect on all-cause mortality. *P. aeruginosa* combined with moderate- or high-risk comorbidities posed an increased risk of mortality. The management of comorbidities may be a critical target during the treatment of *P. aeruginosa* infection in bronchiectasis.

**KEY MESSAGE:**
1. *P. aeruginosa* increased the risk of frequent hospitalizations; however, it had no independent impact on all-cause mortality.
2. *P. aeruginosa* combined with moderate- or high-risk comorbidities posed an increased risk of mortality.
3. The management of comorbidities may be a critical target during the treatment of *P. aeruginosa* infection in bronchiectasis.

## Introduction

Non-cystic fibrosis bronchiectasis (hereafter referred to as bronchiectasis) is a chronic progressive bronchus or bronchiole dilation due to complex interactions between recurrent infection, unbalanced immune regulation, impaired mucociliary clearance, and progressive airway structure damage or obstruction [1]. The estimated prevalence of bronchiectasis is 52.3 cases per 100,000 in the United States [2] and 67 cases per 100,000 in Germany [3], and the prevalence increases each year by 8.7% [4]. The crude annual mortality rate of bronchiectasis varies between 2% and 10% [5–7], which continues to pose a heavy disease burden on both developing and developed countries.

*Pseudomonas aeruginosa* (*P. aeruginosa*) tops the list of organisms isolated from sputum in patients with bronchiectasis, either during stable state or exacerbation [8,9]. It is usually considered to be highly responsible for the poor clinical outcomes of bronchiectasis. As bronchiectasis is a heterogeneous disease, the aetiology, microbial spectrum, and disease severity vary under different geographic and economic background [10]. Some studies have suggested that chronic infection with *P. aeruginosa* independently affects clinical outcomes, including hospitalizations, exacerbations [11,12] and mortality [12,13], in bronchiectasis, whereas other studies have drawn contrasting conclusions [5,14,15]. It remains controversial whether *P. aeruginosa* has an independent effect on clinical outcomes or is simply a marker of disease severity.

In China, bronchiectasis has long been neglected, and studies regarding clinical profiles and the impact of *P. aeruginosa* infection on its prognosis are scanty, mainly concentrated in two developed metropolises [12,16]. Therefore, we aimed to explore the clinical profiles of *P. aeruginosa* infection in bronchiectasis exacerbation and its effect on all-cause mortality, future hospitalizations, and exacerbations in a large tertiary hospital in central-southern China.

## Patients and methods

### Study design and participants

**Figure 1.**
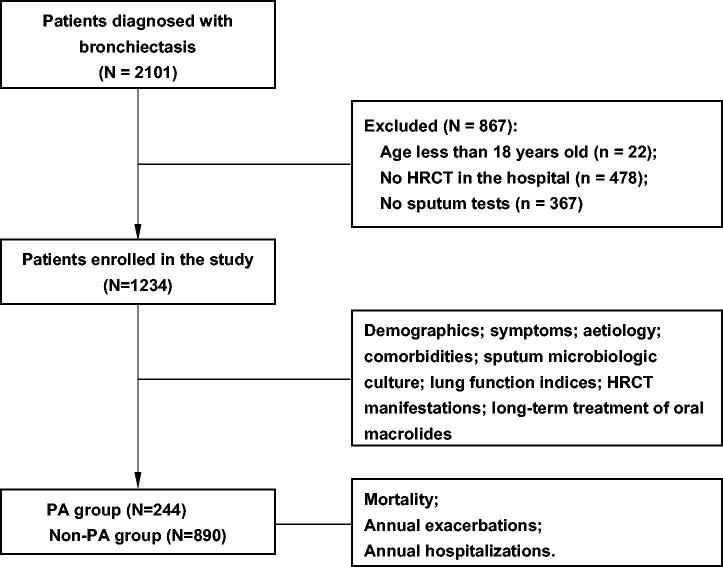
Flow chart of patient enrolment and analysis. HRCT: high-resolution computed tomography; PA: *Pseudomonas aeruginosa*.

This prospective cohort study collected data on patients with bronchiectasis exacerbation between 2013 and 2019 at the Second Xiangya Hospital of Central South University. A flowchart of patient enrolment is displayed in Figure 1. Consecutive adult patients with a diagnosis of bronchiectasis based on high-resolution computed tomography (HRCT) scans and presenting corresponding respiratory symptoms attributable to bronchiectasis exacerbation were enrolled according to the guidelines [17]. Patients were excluded if they lacked HRCT scan results or records of sputum culture in the hospital. Only the first medical records of patients with multiple admissions were reviewed for our analysis. This study was approved by the institutional review board of the Second Xiangya Hospital in China (luoh202006), and informed consent was obtained from all participants.

### Data collection

Data on demographics (age, sex, occupation, height, and weight), disease course, symptoms, aetiology, comorbidities, forced expiratory volume in the first second (FEV1% predicted), radiologic findings, long-term treatment of macrolides, and Medical Research Council (MRC) dyspnoea score were collected. The aetiology and diseases associated with bronchiectasis were identified according to Spanish guidelines [18].

The Bronchiectasis Aetiology Comorbidity Index (BACI) [19], which is a sum score (range, 0–55) of 13 weighted diseases, was used to assess comorbidities, where higher scores denote an increasing burden of comorbidities specified in bronchiectasis. Patients were classified into the following tertiles: low-risk comorbidities (for patients with a score of 0), moderate-risk comorbidities (for patients with scores ≥1 and <6), and high-risk comorbidities (for patients with a score ≥6).

The radiological severity of bronchiectasis was assessed using a modified Reiff score (range,1–18), which rates the number of involved lobes (of a total of six, with the lingula considered separate) and the degree of dilatation (tubular = 1, varicose = 2, and cystic = 3), as in previous bronchiectasis studies [11,20].

The Bronchiectasis Severity Index (BSI) (age, body mass index [BMI], dyspnoea, exacerbation, FEV1% predicted, microbial colonization, radiological extension, and range of 0–26) was used to assess bronchiectasis severity as mild (score range, 0–4), moderate (score range, 5–8), and severe (score range, 9–26). As microbial colonization status was unavailable in our study, we used data on baseline sputum microbial culture in the hospital as an alternative to microbial colonization.

### Sputum microbiology and group assignment

All microbiological tests were performed on spontaneous sputum samples. Sputum samples were considered eligible if they contained more than 25 leukocytes and less than 10 squamous cells per low-powered field (10 × 10). Specific microorganism infection was considered if sputum cultures were positive on one or more occasions and accorded with clinical physician judgement.

We assigned patients into the following two groups: (1) the PA group for those with *P. aeruginosa* isolation from sputum and (2) the non-PA group for those without *P. aeruginosa* isolation from sputum.

### Follow-up and clinical outcomes

Patients were followed up after discharge *via* specifically designed telephone interviews every 1–3 months. Follow-up was completed on 30 June 2020. There was a median follow-up duration of 16 (1–36) months, in which the overall number of exacerbations and hospitalizations due to exacerbations during the follow-up period were recorded, and mortality was assessed.

The clinical outcomes in this study were all-cause mortality, frequency of annual exacerbations, and frequency of annual hospitalizations due to exacerbations.

An exacerbation of bronchiectasis was defined as at least three of the following symptoms: cough frequency, sputum volume and/or consistency, sputum purulence, dyspnoea and/or exercise tolerance, fatigue and/or malaise, and haemoptysis; where the condition deteriorated for at least 48 h, and beyond the daily variation range, a change in treatment was required [21]. Patients were considered to have frequent exacerbations if there were two or more annual exacerbations (total number of exacerbations divided by follow-up years) and frequent hospitalizations if there were one or more annual hospitalizations (total number of hospitalizations divided by follow-up years).

### Statistical analysis

Descriptive statistics of demographic and clinical variables are presented as median (interquartile range) or counts (n) and proportions (%), unless otherwise stated. Data comparisons were applied between the PA and non-PA groups. The chi-squared test was used for categorical data. The student’s *t*-test was used for continuous variables showing normal distributions and the Kruskal–Wallis test for those with non-normal distributions. Unless stated otherwise, the significance level was set at *p* < .05.

Kaplan–Meier curves were constructed to illustrate survival data, and Cox proportional hazard regression analysis was used for univariate and multivariable mortality analysis to estimate hazard ratios (HRs) and their 95% confidence intervals (CIs). Similarly, univariate and multivariate logistic regression analyses were performed to identify variables associated with bronchiectasis hospitalizations and exacerbations, and odds ratios (ORs) and their 95% CIs were calculated. We included the following baseline variables when studying potential in the univariate analyses: sex; age; BMI; smoking habit; symptoms of haemoptysis; exacerbations in the previous year; MRC dyspnoea score; Reiff score; FEV1% predicted; BACI score; aetiology and associated diseases (unknown aetiology, chronic respiratory disease; connected tissue disease, post-infectious); long-term oral macrolides; sputum microbiologic culture (*P. aeruginosa, Acinetobacter baumannii, Klebsiella pneumoniae, Escherichia coli, Haemophilus influenzae, Staphylococcus aureus, Candida,* and *Aspergillus fumigatus*); BSI stage; status of PA; and comorbidities (only for survival analysis). Each variable was initially tested individually before we added all variables that showed a univariate association (*p* < .10) to the multivariable model, except for sex and age, which had to appear in both models. Backward stepwise selection (likelihood ratio) (*p*_in_ < 0.05 and *p*_out_ > 0.10) was used to determine the factors associated with mortality, hospitalizations, and exacerbations. The Hosmer–Lemeshow goodness-of-fit test was performed to assess the overall fit of the final model. All data were analysed using SPSS version 26.0 (SPSS Inc., Chicago, IL, USA).

## Results

### Patients characteristics

We recruited 1,234 patients in this study (Table 1). Overall, there were slightly more men than women (52% versus 48%). The median age was 62.5 years. Of the patients, 94.2% were over 40 years of age and 39.5% over 65 years of age. Two fifths of them had a history of smoking. The median disease course was eight years. Regarding aetiology of bronchiectasis, 407 patients (33%) had post-infection, 355 (28.8%) chronic respiratory disease (chronic obstructive pulmonary disease [COPD], asthma, and pneumoconiosis), 73(5.9%) connective tissue disease, and 254 (20.6%) unknown aetiology (Table 1). A total of 428 patients (34.7%) had low-risk comorbidities (BACI score of 0), 495 (40.1%) moderate-risk comorbidities (BACI score of 1–6), and 311 (25.2%) high-risk comorbidities (BACI score > 6). The majority of the patients (84.7%) were in the moderate or severe stage according to BSI score.

**Table 1.** General characteristics of bronchiectasis patients.

	Total	PA group	Non-PA group	*p*-value
Patients	1234 (100%)	244 (19.8%)	990 (81.2%)	–
Demographics				
Male	642 (52%)	106 (43.4%)	536 (54.1%)	.03
Age (years)	62.5 (53.0–70.0)	62 (53–68)	63 (53–70)	.532
Body mass index (kg*m^–2^)	20.83 (18.29–23.60)	20.03 (17.63–22.66)	21.05 (18.43–23.88)	<.001
Smokers and ex-smokers	487 (39.5%)	84 (34.4%)	403 (40.7%)	.072
Clinical status				
Disease course (years)	8 (1–20)	13 (5–30)	5.5 (1–20)	<.001
Haemoptysis		112 (45.9%)	132 (54.1%)	.077
MRC dyspnoea score	2 (0–3)	2 (1–2)	2 (0–3)	.641
Annual exacerbations	2 (1–3)	1 (1–2)	1 (1–2)	.397
Annual hospitalizations	0 (0–1)	1 (0–2)	0.5 (0–1)	<.001
Aetiology and associated diseases				
Post-infectious	407 (33.0%)	80 (32.8%)	327(33.0%)	.942
Associated with chronic respiratory disease	355 (28.8%)	76 (31.1%)	280(28.3%)	.376
Associated with connected tissue disease	73 (5.9%)	9 (3.7%)	64(6.5%)	.128
Unknown aetiology	254 (20.5%)	59 (24.2%)	195 (19.7%)	.133
Comorbidities				
Chronic obstructive pulmonary disease	346 (28.0%)	70 (28.7%)	276 (27.9%)	.801
Asthma	137 (11.1%)	29 (11.9%)	108 (10.9%)	.733
Pulmonary hypertention	107 (8.7%)	40 (16.4%)	67 (6.8%)	<.001
Ischaemic cardiomyopathy	134 (10.9%)	17 (7.0%)	117 (11.8%)	.029
Diabetes	100 (8.1%)	21 (8.6%)	79 (8.0%)	.748
Connective tissue disease	110 (8.9%)	20 (8.2%)	90 (9.1%)	.661
BACI score	3 (0–6)	3 (0–7)	3 (0–6)	.186
BACI score = 0	428 (34.7%)	78 (32.0%)	350 (35.4)	–
BACI score 1–6	495 (40.1%)	98 (40.2%)	397 (40.1%)	–
BACI score over 6	311 (25.2%)	68 (27.9%)	243 (24.5%)	–
Lung function				
FEV1(% predicted)	53.12 (36.47–71.27)	45.5 (31.5–65.1)	52.9 (35.8–72.1)	<.001
FEV1(% predicted) ＜50%	555 (45.0%)	74 (30.3%)	195 (19.7%)	<.001
Radiology				
Cystic bronchiectasis	244 (19.8%)	154 (63.1%)	90 (36.9%)	<.001
Involved lung Lobes	3 (0–6)	6 (3–6)	2 (1–6)	<.001
Reiff score	6 (1–18)	12(6–18)	4 (2–9)	<.001
BSI stage				<.001
Mild	189 (15.3%)	0 (0%)	189 (19.1%)	–
Moderate	604 (59.0%)	0 (0%)	189 (19.1%)	–
Severe	441 (35.7%)	46 (18.9%)	558 (56.4%)	–
Treatment	3 (0–22)	198 (81.1%)	243 (24.5%)	–
Long-term oral macrolides	54 (4.4%)	18 (7.4%)	36 (3.6%)	.011
Follow up				
More than 2 annual exacerbations	149 (12.1%)	43 (17.6%)	106 (10.7%)	.002
More than 1 annual hospitalization	226 (18.3%)	65 (26.6%)	161 (16.3%)	<.001
Mortality rate (deaths per person*year of observation)	188/2204	45/444	143/1760	–

Data are presented as n (%) or median (interquartile range) unless otherwise stated. PA: *Pseudomonas aeruginosa*; MRC: Medical Research Council; FEV1: forced expiratory volume in 1 s; BACI: Bronchiectasis Aetiology Comorbidity. Index; BSI: Bronchiectasis Severity Index.

### Microbiology

Overall, 479 (38.8%) patients had positive sputum-test results for pathogenic microorganisms. Sixty-three patients (5.1%) were co-infected with *P. aeruginosa* and other microorganisms. As regards bacteria, *P. aeruginosa* was the most common positive pathogen in 244 (19.8%) patients, followed by *Acinetobacter baumannii* in 48 (3.9%), *Klebsiella pneumoniae* in 47 (3.8%), *Escherichia coli* in 21 (1.7%), and *Haemophilus influenzae* in 13 (1.0%). Regarding fungi, *Candida* was more common (*n* = 108, 8.8%) than *Aspergillus fumigatus* (*n* = 57, 4.6%). The details are shown in Table 2.

**Table 2.** Microbiological characteristics of subjects with bronchiectasis.

Pathogens	Numbers (N)	Percentage#	Percentage*
Total#	1234	–	–
Total*	496	40.2%	–
Bacteriologic			
* Pseudomonas aeruginosa*	244	19.8%	49.2%
* Acinetobacter baumannii*	48	3.9%	9.7%
* Klebsiella pneumoniae*	47	3.8%	9.5%
* Stenotrophomonas maltophilia*	24	1.9%	4.8%
* Escherichia coli*	21	1.7%	4.2%
* Mycoplasma pneumoniae*	24	1.9%	4.8%
* Staphylococcus aureus*	17	1.4%	3.4%
* Haemophilus influenzae*	13	1.1%	2.6%
* Mycobacterium tuberculosis*	13	1.1%	2.6%
* Enterobacter cloacae*	9	0.7%	1.8%
* Legionella pneumophila*	7	0.6%	1.4%
* Serratia marcescens*	5	0.4%	1.0%
Mycological			
* Candida*	108	8.8%	21.8%
* Aspergillus fumigatus*	57	4.6%	11.5%
Other species	27	2.20%	5.4%

^#^Indicates the patients included in this study. *Indicates the patients who had positive culture in sputum. Other species include Streptococcus pneumoniae, Enterococcus faecium, Proteus mirabilis, Alcaligenes, Malodorous pseudomonas, Acinetobacter roffei, Moraxella catarrhalis, Enterobacter aerogenes, Candida tropicalis and Mucor.

### Differences in characteristics between PA group and non-PA group patients

Compared to those in the non-PA group (Table 1), patients in the PA group had a higher female ratio (56.6% versus 45.9%), lower BMI (20.04[17.63–22.66] kg/m^2^ versus 21.05 [18.43–23.88] kg/m^2^, *p* < .001), and longer disease course (13 [5–30] years versus 5.5 [1–20] years). As regards etiology, there was no significant difference between the PA and non-PA groups. In terms of comorbidities, patients in the PA group had a higher proportion of pulmonary hypertension (16.4% versus 6.8%, *p* < .001) and a similar BACI score to those in the non-PA group. Patients in the PA group had poorer lung function (lower FEV1% predicted), more cystic bronchiectasis, more lung lobes involved, and more severe disease stages according to BSI score. Patients in the PA group also had more frequent future annual exacerbations (17.6% versus 10.1%, *p* = .002) and hospitalizations (26.6% versus 16.3%, *p* < .001).

### All-cause mortality

Overall, 188 patients (15.2%) died during the follow-up period. The all-cause mortality was 18.4% (45 of 244 patients) in the PA group and 14.4% (143 of 990 patients) in the non-PA group.

**Figure 2.**
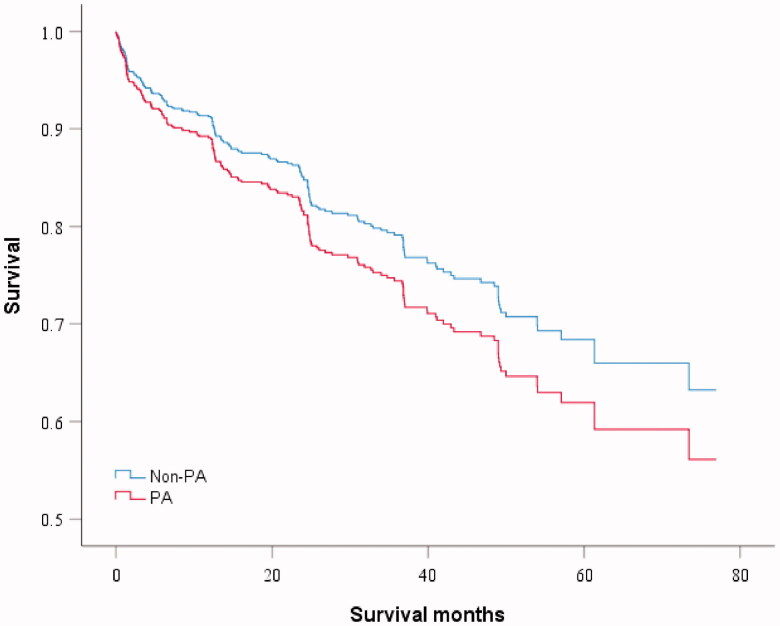
Kaplan–Meier log-rank test survival curve and univariate analysis for mortality. Pseudomonas aeruginosa (PA) versus non-PA. Hazard ratio for death for PA infection was 1.26 (95% CI 0.90–1.76, *p* = .176) in Cox proportional hazard regression analysis.

The univariate analysis of the influence of *P. aeruginosa* on mortality showed a higher risk trend but without statistically significant differences (HR: 1.26, 95% CI: 0.90–1.76, *p* = 0.176) (Figure 2). Multivariate analysis showed that age (HR: 1.03, 95% CI: 1.02–1.05, *p* < .001), BACI score (HR: 1.06, 95% CI: 1.03–1.10, *p* < .001), and *Acinetobacter baumannii* (HR: 1.96, 95% CI: 1.21–3.18, *p* = .006) contributed independently to a higher risk of mortality, whereas higher BMI (HR: 0.93, 95% CI: 0.90–0.97, *p* = .001) had a protective effect against mortality (Table 3). In the second multivariate analysis model of composite scores, BSI stage was associated with mortality.

**Figure 3.**
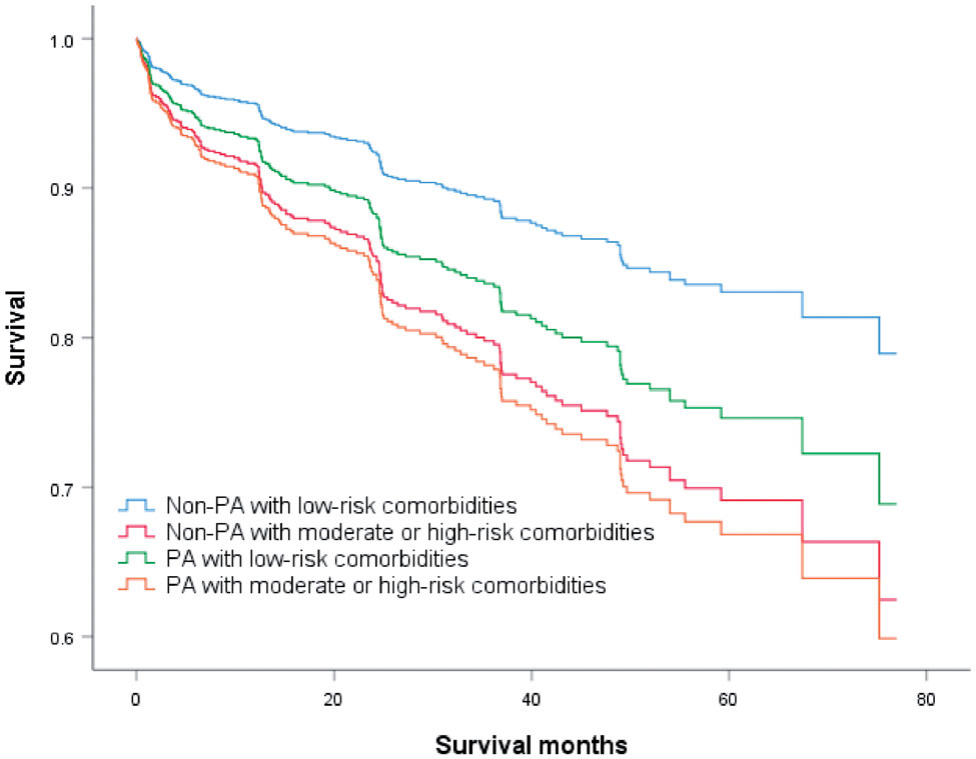
Kaplan–Meier log-rank test survival curve. Comparison between four subgroups: non-Pseudomonas aeruginosa (PA) with low-risk comorbidities; non-PA with moderate or high-risk comorbidities; PA with low-risk comorbidities; PA with moderate or high-risk comorbidities.

**Table 3.** Univariate and multivariate Cox regression analysis of factors associated with mortality.

Variables	Crude HR (95% CI)	*p* value	Adjusted HR (95% CI)^a^	*p* value	Adjusted HR (95% CI)^b^	*p* value	Adjusted HR (95% CI)^c^	*p* value
*Pseudomonas aeruginosa*	1.26 (0.90–1.76)	.176	–	–	–	–	–	–
Female vs male	0.90 (0.67– 1.19)	.452	1.09 (0.74–1.60)	.663	1.12 (0.78–1.62)	0.535	1.01 (0.69–1.48)	.948
Age (years)	1.04 (1.03–1.06)	<.001	1.03 (1.02–1.05)	<.001	–	–	1.04 (1.02–1.05)	<.001
BMI (kg*m^–2^)	0.92 (0.89–0.96)	<.001	0.93 (0.90–0.97)	<.001	–	–	0.93 (0.90–0.97)	<.001
Smoking vs non-smoking	1.31 (0.98–1.74)	.066	1.18 (0.80–1.73)	.401	1.21 (0.84–1.75)	0.311	1.19 (0.82–1.74)	.355
Haemoptysis	0.69 (0.51–0.94)	.018	0.83 (0.60–1.14)	.251	0.77 (0.56–1.06)	0.104	0.81 (0.59–1.12)	.198
Exacerbations in the previous year	1.03 (0.94–1.13)	.496	–	–	–	–	–	–
MRC dyspnoea score	0.95 (0.84–1.07)	.376	–	–	–	–	–	–
Reiff score	1.01 (0.98–1.03)	.675	–	–	–	–	–	–
FEV1% predicte*d* <50% versu*s* ≥ 50%	1.12 (0.84–1.50)	.427	–	–	–	–	–	–
BACI score	1.09 (1.06–1.12)	<.001	1.06 (1.03–1.10)	<.001	1.08(1.06–1.12)	<0.001	–	–
Aetiology and associated diseases							–	–
Unknown aetiology	1.00 (reference)	–	1.00 (reference)	–	1.00 (reference)	–	1.00 (reference)	–
Associated with chronic respiratory disease	1.51 (0.97–1.36)	.071	0.91 (0.56–1.46)	.683	0.88 (0.54–1.42)	0.594	1.00 (0.63–1.60)	.996
Associated with connected tissue disease	1.19 (0.59–2.39)	.623	0.99 (0.49–2.03)	.993	0.83 (0.41–1.68)	0.600	0.98 (0.48–2.02)	.960
Post-infectious	1.47 (0.95–2.27)	.085	1.34 (0.86–2.10)	.198	1.26 (0.81–1.96)	0.313	1.50 (0.96–2.34)	.075
Long-term oral macrolides	0.74 (0.30–1.79)	.497	–	–	–	–	–	–
*Acinetobacter baumannii*	2.84 (1.77–4.57)	<.001	1.96 (1.21–3.18)	.006	–	–	–	–
*Klebsiella pneumoniae*	1.43 (0.76–2.70)	.271	–	–	–	–	–	–
*Escherichia coli*	0.84 (0.27–2.63)	.765	–	–	–	–	–	–
*Haemophilus influenzae*	0.713 (0.1–5.09)	.736	–	–	–	–	–	–
*Staphylococcus aureus*	2.55 (1.13–5.75)	.024	–	–	–	–	–	–
*Candida*	2.27 (1.54–3.33)	<.001	1.30 (0.85–1.98)	.226	–	–	–	–
*Aspergillus fumigatus*	1.33 (0.70–1.52)	.38	–	–	–	–	–	–
BSI stage								
Mild	1.00 (reference)	–	–	–	1.00 (reference)	–	–	–
Moderate	1.80 (1.07–3.03)	.026	–	–	1.75 (1.04–2.95)	0.035	–	–
Severe	2.57 (1.53–4.33)	<.001	–	–	2.36 (1.40–3.98)	0.001	–	–
Status of PA and comorbidities								
Non-PA with low-risk comorbidities	1.00 (reference)	–	–	–	–	–	1.00 (reference)	–
Non-PA with moderate or high-risk comorbidities	2.46(1.62–3.74)	<.001	–	–	–	–	1.93 (1.26–2.95)	.003
PA with low-risk comorbidities	1.55(0.70–3.40)	.28	–	–	–	–	1.56 (0.71–3.44)	.268
PAwith moderate or high-risk comorbidities	2.77(1.69–4.55)	<.001	–	–	–	–	2.11 (1.28–3.48)	.003

HR: Hazard ratio; CI: confidence interval; BMI: body mass index; PA: Pseudomonas aeruginosa; MRC: Medical Research Council; FEV1: forced expiratory volume in 1 s; BSI: Bronchiectasis Severity Index; BACI: Bronchiectasis Aetiology Comorbidity Index. Data are shown as estimated HRs (95% CIs) of the explanatory variables.

^a^Initial multivariable model comprised gender, age, BMI, smoking habit, haemoptysis, BACI score, aetiology and associated diseases, *Acinetobacter baumannii*and *Candida*. Final multivariable model was adjusted for age, BMI, BACI score, *Acinetobacter baumannii*.

^b^Initial multivariable model comprised gender, smoking habit, haemoptysis, BACI score, aetiology and associated diseases and BSI stage. Final multivariable model was adjusted for BACI score and BSI stage.

^c^Initial multivariable model comprised gender, age, BMI, smoking habit, haemoptysis, aetiology and associated diseases and status of PA and comorbidities. Final multivariable model was adjusted for age, BMI and status of PA and comorbidities.

We hypothesised that the association of *P. aeruginosa* with mortality may be comorbidity-dependent. Based on the condition of the *P. aeruginosa* infection andBACI score, we reclassified the patients into four subgroups as follows: (1) non-PA with low-risk comorbidities (*n* = 350); (2) non-PA with moderate- or high-risk comorbidities (*n* = 640); (3) PA with low-risk comorbidities (*n* = 78); and (4) PA with moderate- or high-risk comorbidities (*n* = 166). The survival curves of the four subgroups are shown in Figure 3. In the third multivariable analysis model of subgroups (Table 3), *P. aeruginosa* with low-risk comorbidities had no impact on mortality (HR: 1.56, 95% CI: 0.71–3.44, *p* = 0.268). Moderate- or high-risk comorbidities increased mortality risk (HR: 1.93, 95% CI: 1.26–2.95, *p* = .003), and the effect was magnified in the presence of *P. aeruginosa* (HR: 2.11, 95% CI: 1.28–3.48, *p* = .003).

### Future exacerbations and hospitalizations

Of the 1,234 patients, 494 (40.0%) experienced frequent future exacerbations (two or more future annual exacerbations), and 258 (20.9%) had frequent future hospitalizations (more than one future annual hospitalization). Multivariate analysis indicated that *P. aeruginosa* did not increase the risk of frequent future exacerbations (odds ratio [OR]: 1.15, 95% CI: 0.83–1.59, *p* = .400) (Table 4); however, it indicated a higher possibility of frequent hospitalizations (OR: 1.51, 95% CI: 1.08–2.13, *p* = .018) (Table 5). Comorbidities (BACI score) and BSI score also increased the risk of frequent hospitalizations (OR: 1.06, 95% CI: 1.02–1.10, *p* = .003).

**Table 4.** Univariate and multivariate logistic regression analysis of factors associated with frequent exacerbations.

Variables	Crude OR (95% CI)	*p* value	Adjusted OR (95% CI)^a^	*p* value	Adjusted OR (95% CI)^b^	*p* value
*Pseudomonas aeruginosa*	1.47 (1.11–1.95)	.008	1.15 (0.83–1.59)	.400	–	–
Female versus male	1.01 (0.81–1.27)	.907	1.14 (0.87–1.48)	.339	1.14 (0.89–1.45)	.299
Age (years)	1.01 (1.00–1.02)	.153	1.00 (0.99–1.02)	.414	–	–
Body mass index (kg*m^–2^)	0.95 (0.93–0.98)	.001	0.97 (0.94–1.01)	.099	–	–
Smoking vs non-smoking	1.19 (0.94–1.50)	.152	–	–	–	–
Haemoptysis	0.82 (0.65–1.04)	.094	0.96 (0.86–1.07)	.750	1.02 (0.80–1.32)	.865
Exacerbations in the previous year	2.01 (1.79–2.26)	<.001	1.93 (1.72–2.18)	<.001	–	–
MRC dyspnoea score	0.820 (0.65–1.04)	.094	0.96 (0.86–1.07)	.444	–	–
Reiff score	1.03 (1.01–1.04)	.009	1.01 (0.99–1.03)	.485	–	–
FEV1% predicte*d* <50% versu*s* ≥ 50%	1.14 (0.91–1.44)	.252	–	–	–	–
BACI score	1.06 (1.03–1.09)	<.001	1.04 (1.01–1.07)	.015	1.04 (1.01–1.07)	.012
Aetiology and associated diseases						
Unknown aetiology	1.00 (reference)	–	1.00 (reference)	–	1.00 (reference)	–
Associated with chronic respiratory disease	2.38 (1.69–3.34)	<.001	1.60 (1.07–2.38)	.022	1.89 (1.32–2.71)	.001
Associated with connected tissue disease	1.13 (0.65–1.97)	.676	1.13 (0.61–2.09)	.701	1.01 (0.57–1.78)	.982
Post-infectious	1.36 (0.97–1.90)	.075	1.26 (0.87–1.81)	.225	1.26 (0.90–1.78)	.180
Long-term oral macrolides	1.24 (0.70–2.20)	.458	–	–	–	–
*Acinetobacter baumannii*	1.40 (0.78–2.50)	.257	–	–	–	–
*Klebsiella pneumoniae*	0.93 (0.51–1.69)	.805	–	–	–	–
*Escherichia coli*	1.37 (0.58–3.25)	.476	–	–	–	–
*Haemophilus influenzae*	0.78 (0.26–2.33)	.651	–	–	–	–
*Staphylococcus aureus*	1.70 (0.65–4.43)	.279	–	–	–	–
*Candida*	1.50 (1.01–2.22)	.046	1.21 (0.77–1.89)	.405	–	–
*Aspergillus fumigatus*	1.37 (0.80–1.33)	.249	–	–	–	–
BSI stage						
Mild	1.00 (reference)	–	–	–	1.00 (reference)	–
Moderate	1.61 (1.12–2.31)	.01	–	–	1.44 (1.00–2.08)	.052
Severe	2.65 (1.82–3.84)	<.001	–	–	2.31 (1.58–3.37)	<.001

OR: odds-ratio; CI: confidence interval; BMI: body mass index; MRC: Medical Research Council; FEV1: forced expiratory volume in 1 s; BACI: Bronchiectasis Aetiology Comorbidity Index; BSI: Bronchiectasis Severity Index. Data are shown as estimated ORs (95% CIs) of the explanatory variables.

^a^Initial multivariable model comprised gender, age, BMI, smoking habit, exacerbations in the previous years, haemoptysis, mMRC dyspnoea score, Reiff score, BACI score, aetiology and associated diseases, *Pseudomonas aeruginosa* and *Candida*. Final multivariable model was adjusted for exacerbations in the previous year, BACI score and aetiology and associated diseases.

^b^Initial multivariable model comprised gender, haemoptysis, BACI score and aetiology and associated diseases, BSI stage. Final multivariable model was adjusted for BACI score, aetiology and associated diseases and BSI stage.

**Table 5.** Multivariate logistic regression analysis of factors associated with frequent hospitalizations.

Variables	Crude OR (95% CI)	*p* value	Adjusted OR (95% CI)^a^	*p* value	Adjusted OR (95% CI)^b^	*p* value
*Pseudomonas aeruginosa*	1.76 (1.28–2.42)	<.001	1.51 (1.08–2.13)	.018	–	–
Female versus male	1.17 (0.89–1.54)	.276	1.10 (0.75–1.59)	.63	1.16 (0.81–1.66)	.416
Age (years)	1.02 (1.01–1.03)	<.001	1.02 (1.00–1.03)	.005	–	–
Body mass index (kg*m^–2^)	0.97 (0.94–1.01)	.100	0.99 (0.96–1.03)	.716	–	–
Smoking vs non-smoking	1.53 (1.16–2.02)	.003	1.27 (0.87–1.85)	.216	1.39 (1.04–1.85)	.024
Haemoptysis	0.89 (0.67–1.18)	.427	–	–	–	–
Exacerbations in the previous year	1.60 (1.44–1.77)	<.001	1.58 (1.42–1.76)	<.001	–	–
MRC dyspnoea score	0.98 (0.87–1.01)	.702	–	–	–	–
Reiff score	1.01 (0.99–1.03)	.409	–	–	–	–
FEV1% predicte*d* <50% versu*s* ≥ 50%	1.34 (1.02–1.77)	.036	1.21 (0.90–1.64)	.203	–	–
BACI score	1.08 (1.05–1.11)	<.001	1.07 (1.03–1.10)	<.001	1.07 (1.04–1.10)	<.001
Aetiology and associated diseases						
Unknown aetiology	1.00 (reference)	–	1.00 (reference)	–	1.00 (reference)	–
Associated with chronic respiratory disease	2.36 (1.55–3.61)	<.001	1.41 (0.88–2.26)	.159	1.61 (1.02–2.52)	.040
Associated with connected tissue disease	1.57 (0.80–3.01)	.188	1.63 (0.80–3.32)	.181	1.33 (0.67–2.65)	.410
Post-infectious	1.44 (0.93–2.20)	.100	1.30 (0.82–2.06)	.271	1.28 (0.83–1.99)	.269
Long-term oral macrolides	0.75 (0.36–1.55)	.435	–	–	–	–
* Acinetobacter baumannii*	1.13 (0.57–2.24)	.727	–	–	–	–
* Klebsiella pneumoniae*	1.31 (0.67–1.56)	.428	–	–	–	–
* Escherichia coli*	0.89 (0.30–2.66)	.833	–	–	–	–
* Haemophilus influenzae*	0.69 (1.51–3.11)	.625	–	–	–	–
* Staphylococcus aureus*	2.69 (1.02–7.15)	.047	2.31 (0.75–7.07)	.143	–	–
* Candida*	1.76 (1.14–2.72)	.011	1.21 (0.74–1.98)	.446	–	–
* Aspergillus fumigatus*	1.25 (0.67–2.31)	.488	–	–	–	–
BSI stage						
Mild	1.00 (reference)	–	–	–	1.00 (reference)	–
Moderate	1.51 (0.94–2.44)	.088	–	–	1.42 (0.88–2.30)	.154
Severe	2.72 (1.69–4.38)	<.001	–	–	2.52 (1.56–4.07)	<.001

OR: odds-ratio; CI: confidence interval; BMI: body mass index; MRC: Medical Research Council; FEV1: forced expiratory volume in 1 s; BACI: Bronchiectasis Aetiology Comorbidity Index; BSI: Bronchiectasis Severity Index. Data are shown as estimated ORs (95% CIs) of the explanatory variables.

^a^Initial multivariable model comprised gender, age, BMI, smoking habit, exacerbations in the previous years, FEV1% predicted, BACI score, aetiology and associated diseases, *Pseudomonas aeruginosa, Staphylococcus aureus and Candida*. Final multivariable model was adjusted for exacerbations in the previous year, BACI score and aetiology and associated diseases.

^b^Initial multivariable model comprised gender, smoking habit, BACI score, aetiology and associated diseases and BSI stage. Final multivariable model was adjusted for smoking habit, BACI score, aetiology and associated diseases and BSI stage.

## Discussion

To the best of our knowledge, this is the largest Chinese single-center study on clinical profiles and outcomes of *P. aeruginosa* infection in bronchiectasis. Our study demonstrates that *P. aeruginosa* increased the risk of frequent hospitalizations in bronchiectasis but had no independent role in all-cause mortality and frequent exacerbations. Moderate- or high-risk comorbiditiesincreased the risk of all-cause mortality, and the risk could have been magnified by *P. aeruginosa*.

*P. aeruginosa* was the most common organism (19.8%) isolated from the sputum of bronchiectasis patients, which is consistent with the findings of some previous reports [5,16,22] yet different from thoseof others [10,13,14]. The reason for these disparities may be the difference in the definition of *P. aeruginosa* infection, geographic and economic background, and race.

In our study, patients with *P. aeruginosa* had many different clinical profiles from those without *P. aeruginosa*, such as a longer clinical course, poorer lung function, higher rate of pulmonary hypertension, more radiologic involvement, and more frequent future hospitalizations, indicating that *P. aeruginosa* is a marker of disease severity.

The all-cause mortality was 15.2% with a mean follow-up of 16 months in our cohort, which is higher than that reported by Araujo et al. (10.8% in 5 years) and Wang et al. (7.6% in 44 months) [12,14]. The reason for the higher mortality rate in this cohort might have been the complex comorbidities in these patients, considering that 40.1% of the patients had moderate-risk comorbidities and 25.2% high-risk comorbidities. Another reason might have been the severe disease stage of the patients, as the majority of these patients fell into the moderate or severe BSI stage.

The effect of *P. aeruginosa* on the risk of mortality in patients with bronchiectasis is controversial. Several studies have reported that chronic infection with *P. aeruginosa* increases the risk of mortality in bronchiectasis patients [5,13,23]; however, Chalmers et al. [11] and Araujo et al. [14] analysed data from a large cohort of 2,596 patients and found that *P. aeruginosa* had no independent impact on mortality. Previous studies have assessed the impact of certain comorbidities, such as COPD, asthma, and connective tissue disease, without a systematic review of comorbidities [5,6,11,12,14,15]. An international, multicenter cohort analysis of outpatients with bronchiectasis from Europe utilized the BACI score to assess comorbidities comprehensively and demonstrated that the BACI score could predict mortality; nevertheless, this study did not explore the compound effect of comorbidities with other clinically important factors, such as *P. aeruginosa* [19]. In our cohort, we found that the independent predictors of all-cause mortality were age, low BMI and BACI score, and BSI stage but not *P. aeruginosa* infection. In the subgroup analysis, we found that moderate- or high-risk comorbidities increased the risk of all-cause mortality. Furthermore, *P. aeruginosa* magnified the risk of mortality in patients with moderate- or high-risk comorbidities. These data suggest the importance of managing comorbidities in all bronchiectasis patients, especially in cases of moderate- or high-risk comorbidities combined with *P. aeruginosa* presence.

Our study showed that *P. aeruginosa* infection was independently associated with frequent hospitalizations due to exacerbations, which is consistent with the findings of a previous study [11,12,14,15,24]. On the other hand, our study found no independent association of *P. aeruginosa* infection with frequent exacerbations, contradicting the findings from the previous study [11,12,14,15]. As oral antibiotics and mucolytic are available in many places in China [25], bronchiectasis patients often take self-prescribed antibiotics irregularly to suppress the symptoms instead of consulting doctors, which potentially underestimates the exacerbation rates and may be the reason we failedto observe the independent effect of *P. aeruginosa* on frequent exacerbations. Hospitalizations signify a more severe disease condition, heavier disease burden, and high expenditure, despite different admission criteria in different areas. The eradication of *P. aeruginosa* colonization may reduce hospitalization and disease burden in the bronchiectasis population. Recently, the eradication of *P. aeruginosa* has focussed on antibiotic therapy [18,26]. Limited randomised controlled studies have shown that long-term oral macrolides can prevent bronchiectasis exacerbations [27–29]. As bronchiectasis progression is a complex interaction of the host, environment, and microorganisms, interventions for *P. aeruginosa* infection in bronchiectasis are not limited to antibiotics. Host immune regulation, including passive and active immunity, and pulmonary rehabilitation, especially the airway clearance technique, have been utilized in individual treatments [30]. Large randomized controlled trials are still required to clarify the management of bronchiectasis with *P. aeruginosa* infection.

Our study has several limitations. First, this was a prospective study in a single-center tertiary hospital, where we could not define the PA group as chronic *P. aeruginosa* colonization because we were unable to perform subsequent repeated sputum tests to confirm their state of bacterial colonization. It would be valuable to trace multiple cultures in future longitudinal studies to assess chronic *P. aeruginosa* colonization, and large prospective cohort or randomized controlled trials are warranted to further evaluate the influence of *P. aeruginosa* infection on mortality and exacerbations. Second, we described microbiologic features based on sputum culture on account of its availability, which may underestimate microbial diversity, especially in viruses. Metagenomics sequencing of respiratory specimens may provide more insights into microbes in the future [31]. Third, we did not analyse the impact of the *P. aeruginosa* resistance pattern and phenotype/strain type on clinical outcomes. The existence of multidrug-resistant *P. aeruginosa* (MDR-PA) isolates is an emergent health threat worldwide. However, evidence on the association between MDR-PA isolates and bronchiectasis prognosis is limited [16] and MDR-PA needs to be investigated further in future studies. Whereas a cross-sectional study of mucoid *P. aeruginosa* and non-mucoid *P. aeruginosa* in bronchiectasis presented different hospitalizations and exacerbations in the previous year [32], analyses of the prognostic effects of mucoid *P. aeruginosa* and non-mucoid *P. aeruginosa* on bronchiectasis are limited. Therefore, more well-designed prospective studies are required to explore the relationship between *P. aeruginosa* resistance patterns and phenotype/strain type on clinical outcomes. Additionally, we did not consider infections by nontuberculous mycobacterium (NTM) in this study because most patients did not undergo examinations for NTM. NTM infection in bronchiectasis is essential for full assessment, and it is critically important to perform NTM cultures in bronchiectasis in future clinical management.

In conclusion, our study provides a more comprehensive clinical view of the role of *P. aeruginosa* infection in disease severity and burden. *P. aeruginosa* infection in bronchiectasis indicated longer disease courses, poorer lung function, and more severe radiologic involvement. *P. aeruginosa* increased the risk of frequent hospitalizations but had no independent role in all-cause mortality and frequent exacerbations in bronchiectasis. Moderate- or high-risk comorbidities increased the risk of all-cause mortality, and the risk was potentially magnified by the presence of *P. aeruginosa*. Therefore, *P. aeruginosa* infection acts as a marker of disease severity as well as predictor of frequent hospitalizations. The management of comorbidities in bronchiectasis may be a critical target during the treatment of *P. aeruginosa* infections.

## Data Availability

The data is available on Open Science Framework (https://osf.io/qhe3t/?view_only=851a31a564b444b28e8be2e132bddcfd)
